# A Perspective on “Signal Transduction Pathway Mediating Carotid Body Dependent Sympathetic Activation and Hypertension by Chronic Intermittent Hypoxia”

**DOI:** 10.1093/function/zqaf012

**Published:** 2025-03-04

**Authors:** Gary C Sieck

**Affiliations:** Department of Physiology and Biomedical Engineering, Mayo Clinic, Rochester, MN 55905, United States

**Keywords:** Ying-Jie Peng, Y-J, Nanduri, Wang, Hildreth, Prabhakar N.R. Signal

We have all experienced friends and loved ones (and perhaps ourselves) who snore during sleep, then stop moving air into their lungs and struggle with great effort to restore breathing. This pattern of obstructive sleep apnea (OSA) occurs when the upper airway collapses during sleep, thereby blocking the passage of air to the lungs. Yet, breathing efforts persist growing stronger and stronger while lung ventilation continues to be blocked. The absence of alveolar ventilation and gas exchange leads to a decrease in arterial oxygen levels and an increase in carbon dioxide levels. Only with arousal is the airway obstruction cleared, and normal lung ventilation resumed, only to be disrupted again and again throughout the night, leading to chronic intermittent hypoxia (CIH).

The prevalence of OSA and CIH is currently very high (15%-30%) and increasing with the aging population and the worldwide pandemic of obesity and diabetes, which are co-morbidities. With its increasing prevalence, OSA represents a major public health crisis now and into the future, especially considering that OSA and CIH also markedly increase the risk for cardiovascular diseases, including arrythmias, high blood pressure, and heart failure.^1^ This heightened risk for cardiovascular disease is due at least in part to increased sympathetic nerve activity in patients with OSA and CIH. For some time, it has been known that increased sympathetic nerve activity is triggered by the interruption of lung ventilation and gas exchange during OSA, leading to reduced arterial oxygen levels that are sensed via carotid chemoreceptors. Yet, the mechanisms underlying reduced arterial oxygen levels, chemoreception, and increased sympathetic nerve activity are not fully understood.^1^

It is now well established that the sensing of reduced arterial oxygen levels occurs at the carotid chemoreceptors in the carotid bodies located at the bifurcation of the common carotid arteries in the neck. The carotid bodies are logistically situated to sample arterial oxygen levels just after gas exchange by the lungs. Anatomically, the carotid sinus and carotid bodies are quite distinct drawing considerable attention by early anatomists. Because of their gross anatomy, the carotid bodies were initially thought to be small ganglia (ganglion minutum, or ganglion intercaroticum) or glands (glomus caroticum or carotid gland) primarily subserving a motor or endocrine function. One hundred years ago in 1925, the German physiologist Heinrich Hering reported that mechanical or electrical stimulation of the carotid sinus, near the bifurcation of the common carotid arteries, evoked a decrease in blood pressure and a slowing of heart rate, a baroreflex mediated by distinct baroreceptors.^2^ Just a few years later in 2 articles published in 1926^3^ and 1928,^4^ Fernando de Castro, a student of Santiago Ramón y Cajal in Spain, provided an elegant depiction of the microanatomy of the carotid body that clearly showed the sensory innervation of the carotid body by the glossopharyngeal nerve, suggesting a sensory function. At about the same time, Belgian physiologists Jean-François Heymans and his son Corneille Heymans proposed that in addition to the distinct baroreceptor function of the carotid sinus, the carotid body contained chemoreceptors that respond to a decrease in arterial blood oxygen.^5^^,^^6^ Their studies used cross-perfusion of blood between two animals to show that a reduction in the blood levels of oxygen in one animal affected breathing in the second providing clear evidence for peripheral chemoreceptors located in the carotid bodies. In 1938, Corneille Heymans was awarded the Nobel Prize in Physiology or Medicine “for the discovery of the role played by the sinus and aortic mechanisms in the regulation of respiration,” which confirmed the physiological role of the carotid body. His father Jean-François, who died in 1932 before the Nobel Prize was awarded, was not recognized, nor was Fernando de Castro, who arguably made a major contribution to the important discovery of the sensory function of carotid bodies.

Although there are different cell types in the carotid body, it is now well established that glomus or type I cells possess chemoreceptor function. In recent years, considerable progress has been made toward understanding the mechanism of oxygen sensing (sensory transduction) in glomus cells.^7^^,^^8^ In s study published in this issue of *FUNCTION*,^9^ Peng and colleagues extend their previous work exploring sensory transduction in the carotid body by providing compelling converging evidence for a complex signaling cascade for CIH that is triggered by an increase in hydrogen sulfide (H_2_S) levels in carotid body glomus cells. They show that elevated H_2_S then activates olfactory receptor 78 (Olfr78) through persulfidation. Downstream, H_2_S-induced activation of the Olfr78 receptor is then coupled to an increase in cyclic adenosine monophosphate (cAMP) by adenylyl cyclase 3 (Adcy3). The elevated cAMP then activates the calcium channel Cnga2 (encoding cyclic nucleotide–gated channel alpha2 subunit), which increases intracellular calcium levels in glomus cells, leading to a hyperactive carotid body chemoreflex and an increase in sympathetic nerve activity and hypertension. In support of this H_2_S-Olfr78 signaling pathway in glomus cells, Peng and colleagues used molecular approaches involving different mutant mice that blocked signaling at different levels of the signaling cascade. The insight provided by the results of this study will be extremely valuable in developing effective therapeutic interventions to mitigate the increase in sympathetic nerve activity during OSA and CIH. For example, a major finding of this study is that CIH increases cAMP levels in the CB via persulfidation of the Olfr78 receptor and coupling to Adcy3. Identifying the components of this signaling cascade presents several potential pharmacological targets for disrupting this signaling pathway to reduce CIH-induced cAMP generation, thereby desensitizing the carotid body chemoreflex and mitigating the CIH-induced increase in sympathetic nerve activity.
